# Geospatial patterns and socioeconomic determinants of the global acute viral hepatitis burden

**DOI:** 10.3389/fpubh.2025.1581484

**Published:** 2025-06-05

**Authors:** Ke-Jie He, Guoyu Gong

**Affiliations:** ^1^The Quzhou Affiliated Hospital of Wenzhou Medical University, Quzhou People's Hospital, Quzhou, China; ^2^School of Medicine, Xiamen University, Xiamen, China

**Keywords:** acute viral hepatitis, global burden, spatial distribution, decomposition analysis, frontier analysis, targeted interventions

## Abstract

**Background:**

Acute viral hepatitis remains a persistent global health challenge, with significant morbidity and mortality associated with different hepatitis subtypes. Understanding the spatial distribution and underlying drivers of the acute hepatitis burden is crucial for developing targeted interventions.

**Methods:**

This study leveraged data from the Global Burden of Diseases, Injuries, and Risk Factors Study to analyze the geographical disparities and temporal trends in the incidence of acute hepatitis A, B, C, and E. A multifaceted approach was employed, including spatial mapping, decomposition analysis, and frontier analysis, to elucidate the key factors shaping the epidemiological landscape.

**Results:**

The spatial analysis revealed pronounced global heterogeneity in acute viral hepatitis incidence, with the highest rates concentrated in parts of Africa, while Western Europe and North America exhibited significantly lower incidence levels. Decomposition analysis demonstrated that population growth was the leading driver of the increasing global burden across all hepatitis subtypes, particularly in low-SDI and low-middle SDI countries, whereas epidemiological improvements were more prominent in high-SDI countries for hepatitis B and C. Frontier analysis highlighted that countries such as Japan, South Korea, and Singapore, despite their advanced socioeconomic status, still lag behind optimal incidence thresholds, while low-SDI nations like Nepal and Burundi have made remarkable progress relative to their development level. These findings underscore considerable disparities and untapped potential for burden reduction globally.

**Conclusion:**

Our findings confirm substantial spatial variation and development-related disparities in acute viral hepatitis incidence worldwide. The global burden is shaped by a combination of transmission routes, sociodemographic dynamics, and healthcare capacity. Context-specific interventions must be aligned with regional epidemiological profiles—targeting sanitation and vaccination in high-burden areas and optimizing harm reduction and screening in more developed settings. The integration of spatial analysis, decomposition, and frontier benchmarking provides a valuable framework for prioritizing national and global hepatitis control strategies.

## Introduction

Acute viral hepatitis remains a persistent global health challenge, with significant morbidity and mortality associated with the different hepatitis subtypes. Hepatitis A virus (HAV) is primarily transmitted through the fecal-oral route, often via contaminated water sources or food (1). In contrast, hepatitis B virus (HBV) and hepatitis C virus (HCV) are primarily transmitted through exposure to infected body fluids, most commonly during childbirth (2), unsafe sexual practices (3), or injection drug use (4). Hepatitis E virus (HEV) is also transmitted through the fecal-oral route, typically linked to contaminated water sources (5). While acute hepatitis A and E generally resolve with supportive care, HBV and HCV can lead to chronic infections, which are significant public health concerns and major risk factors for the development of cirrhosis and liver cancer (6, 7).

To elucidate the global landscape of acute hepatitis, we leveraged data from the Global Burden of Diseases, Injuries, and Risk Factors Study (GBD), a comprehensive epidemiological assessment of over 300 diseases and injuries across 204 countries and territories. The GBD data provide a robust foundation for analyzing the spatial distribution of acute hepatitis incidence and identifying the key drivers of the observed trends.

In this study, we employed a multifaceted analytical approach to gain insights into the uneven global burden and underlying factors shaping the epidemiology of acute hepatitis. We first mapped the spatial distribution of incidence for each hepatitis subtype to elucidate the geographical disparities. We then conducted a decomposition analysis to quantify the relative contributions of population growth, aging, and epidemiological changes in driving the trends in acute hepatitis burden over time. Additionally, we utilized a frontier analysis to identify the countries with the greatest potential for further reduction in acute hepatitis incidence, given their current socioeconomic development levels. By integrating these complementary analytical techniques, we aim to inform the development of targeted, context-specific interventions to address the persistent challenge of acute viral hepatitis globally.

## Methods

### Data sources

The study leveraged the comprehensive dataset from the 2021 Global Burden of Disease (GBD) study, which evaluated over 300 diseases and 88 risk factors across 204 countries and territories from 1990 to 2021 (8). This robust epidemiological assessment provided the foundation for analyzing the spatial distribution and underlying drivers of the global burden of acute viral hepatitis.

The key data points utilized in this analysis include the incidence rates for each hepatitis subtype (A, B, C, and E), as well as the decomposition of changes in the disease burden over time into the contributing factors of population growth, population aging, and epidemiological changes. The data was further disaggregated by the sociodemographic index (SDI) to enable comparative analyses across the development spectrum, ranging from low SDI to high SDI countries.

We employed advanced analytical techniques, such as Bayesian meta-regression and spatial modelling, to generate the comprehensive estimates of the acute hepatitis burden and its determinants. The input data were derived from various authoritative sources, including scientific literature, national health surveys, and healthcare administrative records, ensuring a robust and comprehensive representation of the global epidemiology of acute viral hepatitis.

### Analytical methods

We employed a multi-method analytical framework to investigate the complex patterns underlying the global burden of acute viral hepatitis.

First, we applied Bayesian meta-regression techniques using the GBD’s DisMod-MR 2.1 tool, which integrates data from a variety of sources, including population surveys, disease registries, and scientific literature, and produces internally consistent estimates of disease metrics. This method adjusts for potential sampling bias and heterogeneity across data inputs, offering robust estimates of incidence and other related measures.

Second, we conducted spatial analyses to map global incidence patterns of each hepatitis subtype. Using geostatistical visualization, we presented national-level estimates of incidence overlaid with color gradients based on the SDI, allowing for comparison across countries at different stages of development. We also examined spatial heterogeneity and clustering to identify regions with disproportionate burdens.

Third, we implemented decomposition analysis to quantify the relative contributions of population growth, population aging, and epidemiological changes to trends in acute hepatitis burden over time. This approach followed a standardized decomposition framework widely used in global health research (9, 10), allowing for the disaggregation of complex temporal dynamics into interpretable components.

Fourth, we performed frontier analysis using stochastic frontier techniques to benchmark countries’ observed incidence against optimal performance expected at their respective SDI levels. This analysis enabled the identification of countries with the greatest potential for improvement and facilitated cross-country comparison of efficiency in reducing acute hepatitis incidence. In our visualizations, black-labeled countries indicate those with the largest gaps relative to the frontier, highlighting their substantial room for progress.

Throughout our analytical journey, we leveraged the versatile R programming language (version 4.2.3) to seamlessly integrate data processing, modeling, and visualization. The detailed methods are provided in Supplementary methods.

### Patient and public involvement

The GBD study is a scientific collaboration that enables the comparison and reproduction of the influence of various health conditions across age, gender, and geographical locations at specific moments. This study has attracted considerable scientific, policy, and public interest on a global scale. In the course of our research, we made use of secondary data from this collaborative undertaking, and we did not have any direct interaction with the participants. The research questions and outcome indicators remained unchanged regardless of patient participation, and patients were not involved in the design or implementation of the study.

## Results

### Uneven global burden and drivers of acute hepatitis: insights for targeted interventions

**Figure 1:**
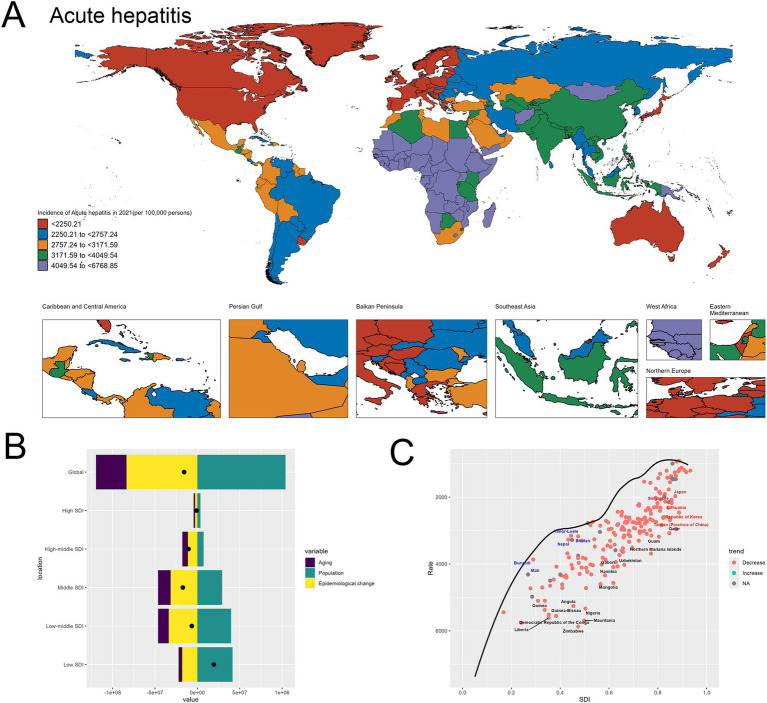
Uneven global burden and drivers of acute hepatitis. **(A)** World map depicting the incidence of acute hepatitis across countries in 2021. **(B)** Decomposition analysis revealing the key drivers of acute hepatitis burden change between 1990 and 2021. **(C)** Frontier analysis identifying countries with the greatest potential for further reduction in acute hepatitis incidence given their socioeconomic development. Red-labeled countries indicate countries where the 2021 rate increased compared to 1990, while blue-labeled countries represent countries where the 2021 rate decreased compared to 1990. The black-labeled countries identify the 15 countries with the largest gaps between their actual 2021 incidence and the optimal (frontier) incidence, suggesting the greatest room for improvement. Conversely, the blue-labeled countries highlight the 5 low-SDI countries that have made the most progress in reducing acute hepatitis incidence relative to their frontier. The red-labeled countries represent the 5 high-SDI countries with the largest gaps to the frontier, underscoring the opportunities for these relatively affluent nations to further minimize the acute hepatitis burden.

The spatial distribution of acute hepatitis incidence in 2021, depicted in the world map (Figure 1A), demonstrates substantial global disparities. Certain regions, such as the Africa, experienced exceedingly high incidence rates exceeding 4,049.54 per 100,000 population. In contrast, many Western European and North American countries exhibited relatively lower incidence, ranging from 2,250.21 to 2,757.24 per 100,000. This geographical heterogeneity underscores the need for targeted, region-specific interventions to address the uneven burden of acute hepatitis worldwide.

The decomposition analysis (Figure 1B) reveals the key drivers behind the change in acute hepatitis burden between 1990 and 2021. Population growth emerged as the dominant contributor to the overall increase, as reflected by the large positive effect of the “Population” factor. While the “Aging” effect was also positive, indicating some improvements in disease management, its magnitude was smaller compared to the population expansion and Epidemiological change. This finding suggests that population dynamics have been a primary force underlying the rising acute hepatitis burden globally during this time period. The specifics are provided in the attached Supplementary material 1.

The frontier analysis (Figure 1C) provides insights into the countries with the greatest potential for further reduction in acute hepatitis incidence given their current socioeconomic development levels. The black-labeled countries identify the 15 countries with the largest gaps between their actual 2021 incidence and the optimal (frontier) incidence, indicating the greatest room for improvement. Conversely, the blue-labeled countries highlight the 5 low-SDI countries that have made the most progress in reducing acute hepatitis incidence relative to their frontier, including Nepal, Mali, Bhutan, Timor-Leste and Burundi. This suggests these nations have been more successful in addressing the disease burden given their development status. On the other end of the spectrum, the red-labeled countries represent the 5 high-SDI countries and regions with the largest gaps to the frontier, such as Japan, Republic of Korea, Lithuania, Taiwan (Province of China) and Singapore. This finding indicates that even highly developed economies have substantial room for improvement in tackling the acute hepatitis challenge, underscoring the need for continued efforts to optimize disease prevention and management strategies across the socioeconomic spectrum. The specifics are provided in the attached Supplementary material 2. Detailed confidence intervals and uncertainty ranges for geographic incidence are provided in Supplementary materials 11–15, offering a comprehensive and statistically rigorous exploration of global hepatitis incidence patterns.

### Uneven global burden and drivers of acute hepatitis A: insights for targeted interventions

**Figure 2:**
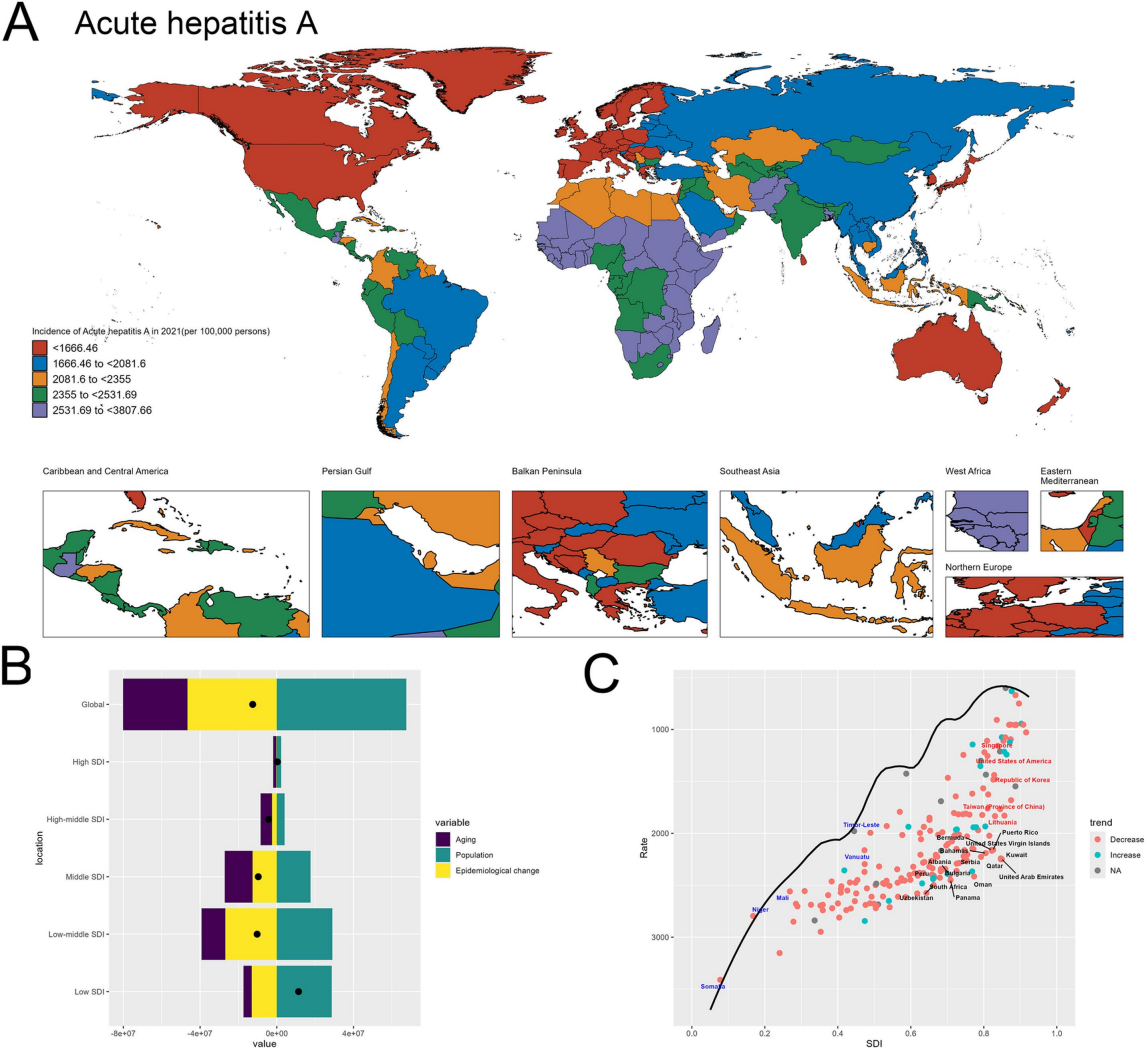
Uneven global burden and drivers of Acute hepatitis A. **(A)** World map depicting the incidence of acute hepatitis A across countries in 2021. **(B)** Decomposition analysis revealing the key drivers of acute hepatitis A burden change between 1990 and 2021. **(C)** Frontier analysis identifying countries with the greatest potential for further reduction in acute hepatitis A incidence given their socioeconomic development.

The Figure 2 provided offer a comprehensive analysis of the global burden of acute hepatitis A, with a particular focus on the spatial distribution of incidence rates and the underlying drivers of change.

The world map in Figure 2A highlights the stark geographical disparities in acute hepatitis A incidence across different regions in 2021. There are many of countries in Africa having exceptionally high incidence rates. In contrast, many Western European nations, including Germany, the United Kingdom, and France, exhibit relatively lower incidence levels ranging (Figure 2A).

The decomposition analysis in Figure 2B provides valuable insights into the key factors driving the changes in acute hepatitis A burden over time. The graph indicates that population growth has been the dominant contributor to the observed overall increase, as evidenced by the substantial positive effect of the “Population” component. While the “Epidemiological change” effect is also positive, suggesting some improvements in disease management, its magnitude is smaller compared to the impact of population expansion. This finding highlights the pivotal role of population dynamics in shaping the trends in acute hepatitis A incidence globally (Figure 2B). The specifics are provided in the attached Supplementary material 3.

In the frontier analysis depicted in Figure 2C, the specific countries that stand out are noteworthy: The black-labeled countries identify the 15 countries with the largest gaps between their actual 2021 acute hepatitis A incidence and the optimal (frontier) incidence, suggesting the greatest potential for improvement. This group includes both low-resource and more developed nations. Furthermore, the red-labeled countries identify the 5 high-SDI countries with the largest gaps to the frontier. This group includes Japan, Singapore, United States of America, Republic of Korea, Taiwan (Province of China) and Lithuania, underscoring the opportunities for these relatively affluent nations and regions to learn from global best practices and enhance their strategies to further minimize the acute hepatitis A burden within their populations. In contrast, the blue-labeled countries highlight the 5 low-SDI countries that have made the most progress in reducing acute hepatitis A incidence relative to their frontier, such as Somalia, Niger, Mali, Vanuatu and Timor-Leste. This suggests these nations have been more successful in allocating their limited resources to tackle the disease, and their experiences could provide valuable lessons for other low-resource settings struggling with high hepatitis A burden. By identifying the countries at both ends of the spectrum, the frontier analysis offers critical insights to guide targeted interventions and the sharing of best practices across the global development landscape to address the persistent challenge of acute hepatitis A (Figure 2C). The specifics are provided in the attached Supplementary material 4.

### Uneven global burden and drivers of acute hepatitis B: insights for targeted interventions

**Figure 3:**
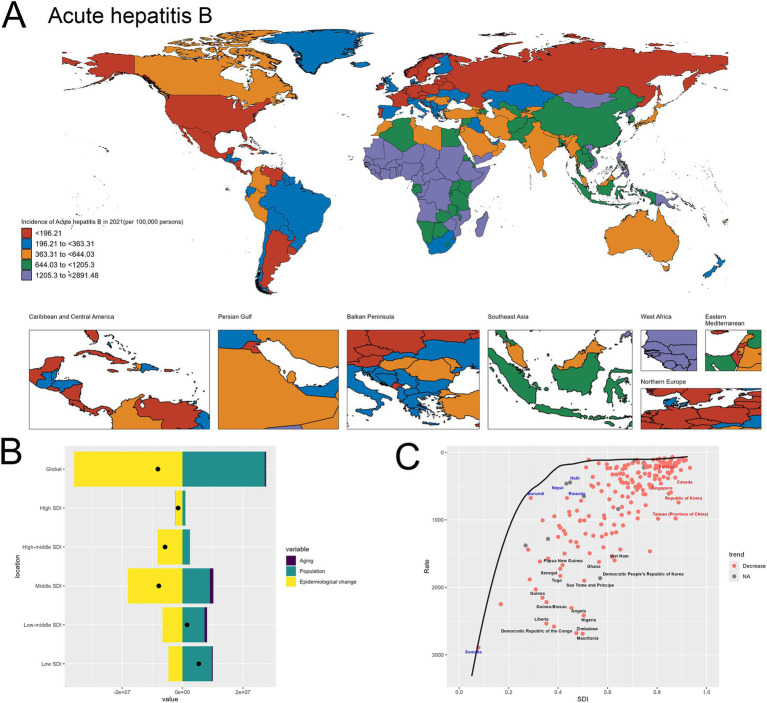
Uneven global burden and drivers of acute hepatitis B. **(A)** World map depicting the incidence of acute hepatitis B across countries in 2021. **(B)** Decomposition analysis revealing the key drivers of acute hepatitis B burden change between 1990 and 2021. **(C)** Frontier analysis identifying countries with the greatest potential for further reduction in acute hepatitis B incidence given their socioeconomic development.

The world map highlights the stark geographical disparities in acute hepatitis B incidence across different regions in 2021. There are many of countries in Africa having exceptionally high incidence rates. In contrast, many high SDI nations, including United States of America, Germany, Mexico and Belarus, exhibit relatively lower incidence levels ranging (Figure 3A).

Upon closer examination of the decomposition analysis, a more nuanced picture emerges regarding the relative contributions of the different drivers of change in acute hepatitis B burden across the development spectrum. For the global level, the decomposition graph shows that the “Epidemiological change” effect, representing improvements in disease prevention and management, plays a more significant role compared to the “Population” effect. This suggests that at the aggregate global level, advancements in the public health response have been a primary factor in shaping the observed changes in acute hepatitis B incidence over time. However, when examining the results across different socioeconomic development levels, a divergent pattern becomes evident. In the high-SDI and high-middle SDI countries, the “Epidemiological change” effect remains the dominant contributor to the changes in acute hepatitis B burden. This indicates that these more developed economies have been able to leverage their stronger healthcare systems and resources to drive improvements in the prevention and control of the disease. In contrast, for the low-SDI and low-middle SDI countries, the “Population” effect emerges as the primary driver of the increase in acute hepatitis B incidence. This finding underscores the critical role of population dynamics, particularly population growth, in shaping the disease burden in resource-constrained settings, where the public health infrastructure and interventions may not have kept pace with the expanding populations. This differentiated analysis across the development spectrum highlights the need for tailored strategies to address acute hepatitis B. While global-level efforts can focus on scaling up effective prevention and treatment programs, targeted approaches are required to address the unique challenges faced by low-resource countries, where population growth remains a significant factor driving the increasing disease burden (Figure 3B). The specifics are provided in the attached Supplementary material 5.

In the frontier analysis depicted in Image C, the red-labeled countries identify the 5 high-SDI countries with the largest gaps to the frontier. This group includes Finland, Singapore, Republic of Korea, Taiwan (Province of China) and Canada, underscoring the opportunities for these relatively affluent nations and regions to learn from global best practices and enhance their strategies to further minimize the acute hepatitis B burden within their populations. In contrast, the blue-labeled countries highlight the 5 low-SDI countries that have made the most progress in reducing acute hepatitis B incidence relative to their frontier, such as Somalia, Burundi, Nepal, Haiti and Rwanda. This suggests these nations have been more successful in allocating their limited resources to tackle the disease, and their experiences could provide valuable lessons for other low-resource settings struggling with high hepatitis B burden (Figure 3C). The specifics are provided in the attached Supplementary material 6.

### Uneven global burden and drivers of acute hepatitis C: insights for targeted interventions

**Figure 4:**
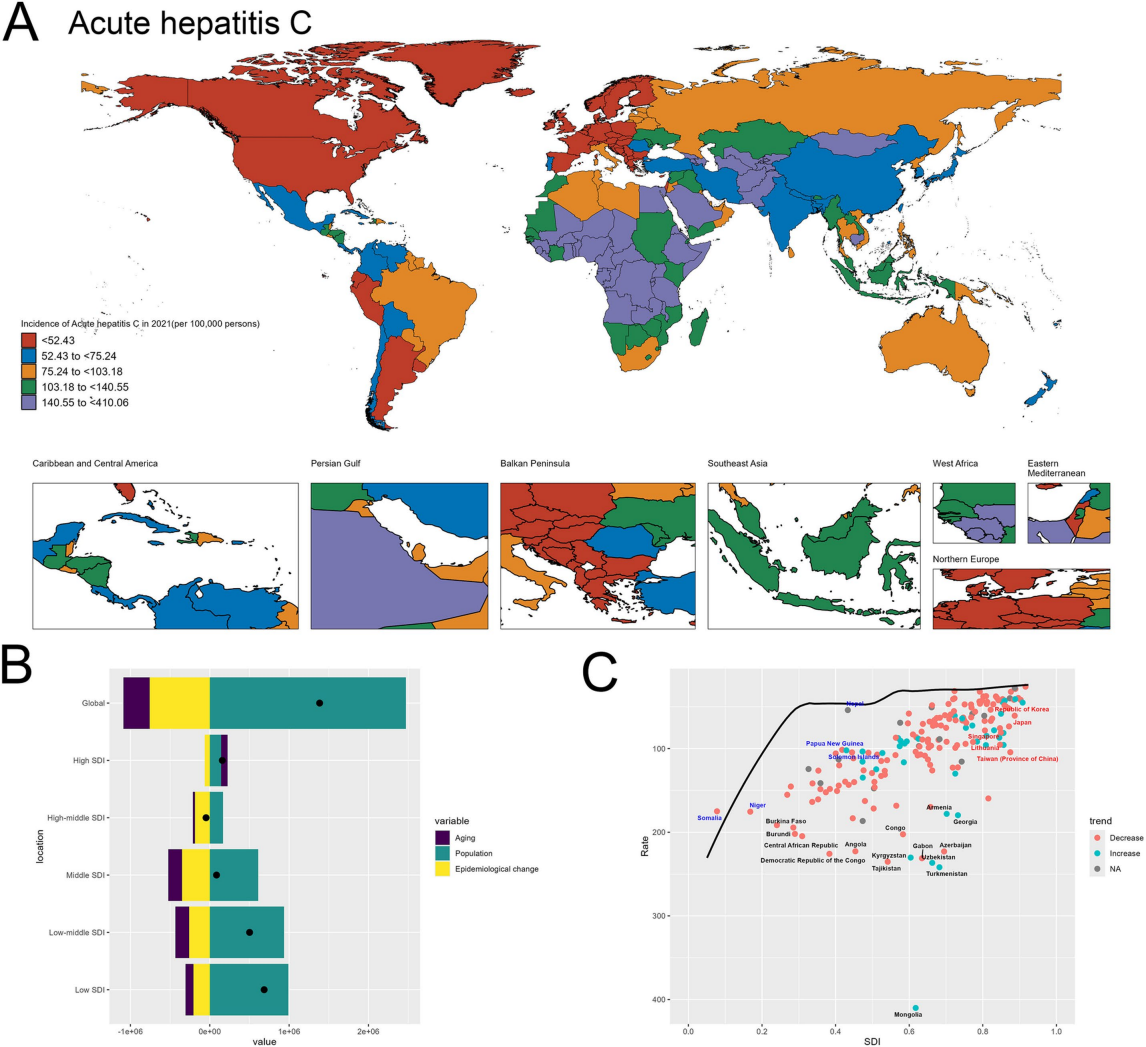
Uneven Global Burden and Drivers of Acute Hepatitis C. **(A)** World map depicting the incidence of acute hepatitis C across countries in 2021. **(B)** Decomposition analysis revealing the key drivers of acute hepatitis C burden change between 1990 and 2021. **(C)** Frontier analysis identifying countries with the greatest potential for further reduction in acute hepatitis C incidence given their socioeconomic development.

Figure 4A presents the world map depicting the incidence of acute hepatitis C in 2021 across different regions. The geographical distribution highlights substantial disparities, with certain areas experiencing remarkably high incidence rates exceeding 140.55 per 100,000 population, such as Mongolia, Egypt, Turkmenistan and Uzbekistan. In contrast, many countries in North America, Europe, and parts of Asia exhibit relatively lower incidence levels (Figure 4A).

At the global level, the decomposition graph shows that the “Population” effect, reflecting improvements in disease prevention and management, is the dominant factor driving the observed changes in acute hepatitis C incidence. This suggests that advancements in public health strategies and interventions have been the primary force shaping the overall trends. However, when examining the results across different socioeconomic development levels, a distinct pattern emerges. In the high-SDI and high-middle SDI countries, while the “Epidemiological change” effect remains the most significant contributor, the relative magnitudes of all the components, including “Population” and “Aging,” are lower compared to the global picture. This indicates that in these more developed economies, the individual drivers have a relatively smaller impact on the overall change in acute hepatitis C burden. In contrast, for the low-SDI and low-middle SDI countries, the “Population” effect emerges as the primary driver of the changes in acute hepatitis C incidence. This finding underscores the critical role of population dynamics, particularly population growth, in shaping the disease burden in resource-constrained settings. In these low-resource nations, the expansion of the population appears to be a dominant factor, potentially outpacing the public health efforts to effectively prevent and manage acute hepatitis C (Figure 4B). The specifics are provided in the attached Supplementary material 7.

In the frontier analysis depicted in Image C, the red-labeled countries identify the 5 high-SDI countries with the largest gaps to the frontier. This group includes Singapore, Republic of Korea, Taiwan (Province of China), Japan and Lithuania, underscoring the opportunities for these relatively affluent nations and regions to learn from global best practices and enhance their strategies to further minimize the acute hepatitis C burden within their populations. In contrast, the blue-labeled countries highlight the 5 low-SDI countries that have made the most progress in reducing acute hepatitis C incidence relative to their frontier, such as Somalia, Niger, Nepal, Papua New Guinea and Solomon Islands. This suggests these nations have been more successful in allocating their limited resources to tackle the disease, and their experiences could provide valuable lessons for other low-resource settings struggling with high hepatitis C burden (Figure 4C). The specifics are provided in the attached Supplementary material 8.

### Uneven global burden and drivers of acute hepatitis E: insights for targeted interventions

**Figure 5:**
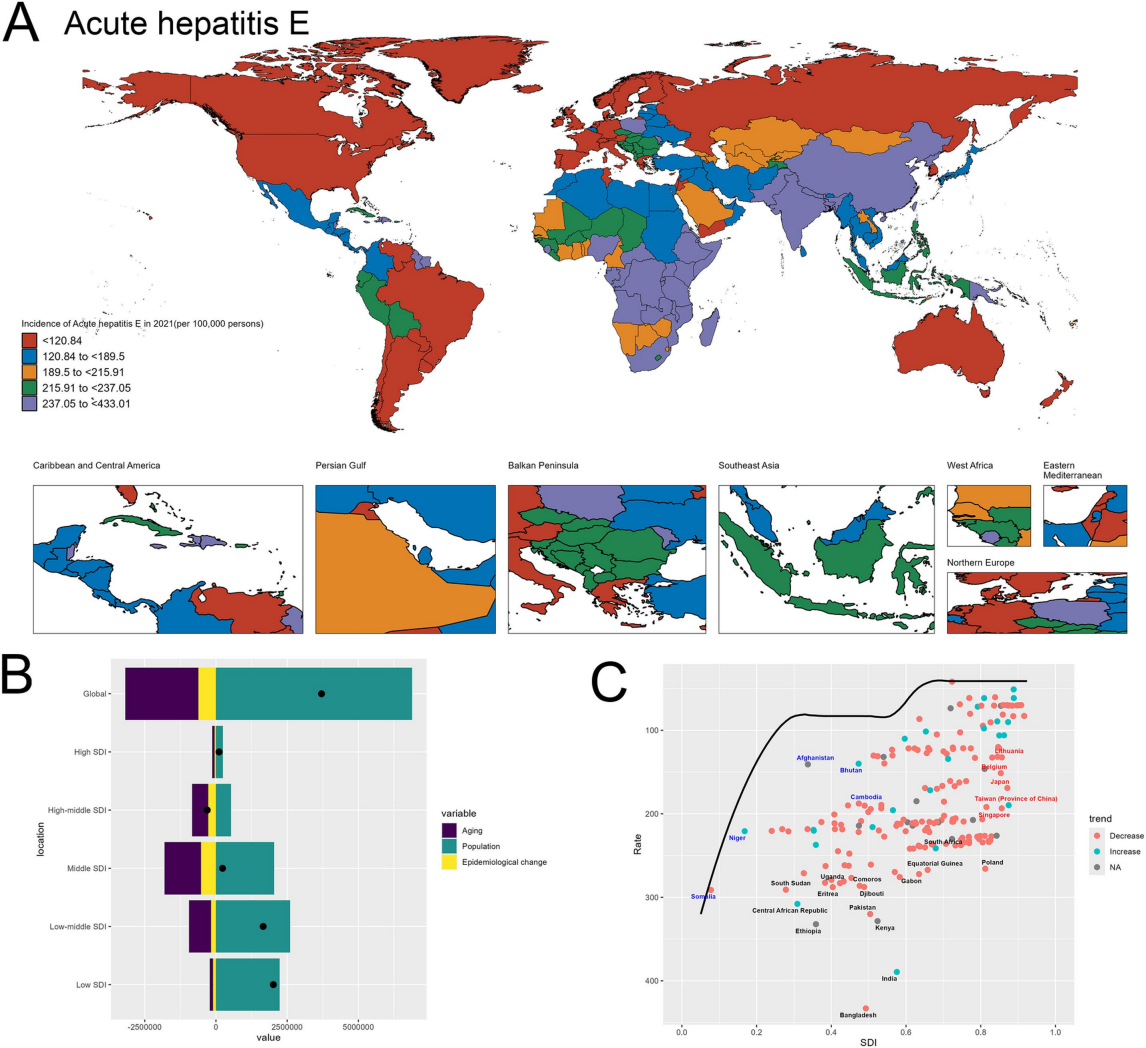
Uneven Global Burden and Drivers of Acute Hepatitis E. **(A)** World map depicting the incidence of acute hepatitis E across countries in 2021. **(B)** Decomposition analysis revealing the key drivers of acute hepatitis E burden change between 1990 and 2021. **(C)** Frontier analysis identifying countries with the greatest potential for further reduction in acute hepatitis E incidence given their socioeconomic development.

Figure 5A shows the world map depicting the incidence of acute hepatitis E in 2021. The geographical distribution highlights significant disparities across regions. Certain areas, such as Bangladesh, India, China and Ethiopia, exhibit exceptionally high incidence rates exceeding 237.05 per 100,000 population, denoted by the dark red shading. In contrast, many countries in North America, Europe display relatively lower incidence (Figure 5A).

The decomposition analysis in Image B provides insights into the key drivers behind the changes in acute hepatitis E burden over time. The graph shows that the “Population” effect, reflecting improvements in disease prevention and management, has been the dominant contributor to the observed changes (Figure 5B). The specifics are provided in the attached Supplementary material 9.

In the frontier analysis depicted in Image C, the red-labeled countries identify the 5 high-SDI countries with the largest gaps to the frontier. This group includes Singapore, Taiwan (Province of China), Japan, Lithuania and Belgium, underscoring the opportunities for these relatively affluent nations and regions to learn from global best practices and enhance their strategies to further minimize the acute hepatitis E burden within their populations. In contrast, the blue-labeled countries highlight the 5 low-SDI countries that have made the most progress in reducing acute hepatitis E incidence relative to their frontier, such as Somalia, Niger, Afghanistan, Bhutan and Cambodia. This suggests these nations have been more successful in allocating their limited resources to tackle the disease, and their experiences could provide valuable lessons for other low-resource settings struggling with high hepatitis E burden (Figure 5C). The specifics are provided in the attached Supplementary material 10.

## Discussion

### Implications of findings

Across all four acute hepatitis subtypes (A, B, C, and E), population growth emerged as the dominant contributor to rising incidence, especially in low and low-middle SDI countries. This universal driver reflects the broader challenge of scaling up health infrastructure in tandem with rapid demographic expansion (11, 12). In many of these regions, such as Africa and India, health systems are under strain, and basic services such as immunization, access to clean water, and infection control are not keeping pace with the needs of a rapidly growing population (13, 14). This demographic pressure not only increases disease exposure but also dilutes the impact of existing public health interventions.

While demographic growth is a shared driver, the transmission modes and structural determinants of each subtype diverge significantly, necessitating subtype-specific policy responses. Hepatitis A and E are predominantly spread via the fecal-oral route (15, 16), and their burden is concentrated in countries lacking access to clean water and adequate sanitation infrastructure. The clustering of high incidence in parts of Africa and South Asia supports the urgent need for WASH (Water, Sanitation, and Hygiene) investment (17, 18). Despite improvements in vaccine availability for hepatitis A (19, 20), the absence of a widely used vaccine for hepatitis E in most endemic countries further complicates control efforts (14). Hepatitis B and C are transmitted through blood and body fluids, with their burden more closely linked to unsafe injection practices, insufficient blood screening, perinatal transmission, and lack of harm reduction services (21–24). These infections are prevalent in both resource-limited and high-income countries, indicating that wealth alone does not guarantee effective control—policy implementation and population-specific outreach remain crucial. This divergence in transmission pathways underscores the importance of contextualized strategies. For example, while WASH improvements are paramount in hepatitis A/E control, hepatitis B/C require robust vaccination, antiviral availability, and harm reduction programs tailored to marginalized populations.

The frontier analysis introduced in this study offers a novel lens to evaluate country performance beyond absolute burden. By benchmarking countries’ incidence rates against SDI-adjusted frontier expectations, we uncovered both exemplars of efficiency and gaps in high-resource settings. Low-SDI countries such as Nepal, Bhutan, Mali, and Somalia consistently outperform expectations, showing how focused, scalable interventions (e.g., targeted vaccination, community-based hygiene education) can yield substantial gains even in constrained environments. For instance, Nepal has implemented a national hepatitis A vaccination program, focusing on high-risk populations and regions with poor WASH conditions (25, 26). Somalia has implemented a comprehensive hepatitis B vaccination program, including both routine childhood immunization and catch-up vaccination for high-risk groups, which has contributed to the observed improvements (27, 28). Nepal and Haiti have also made strides in strengthening their primary healthcare systems and improving access to hepatitis B testing and treatment, demonstrating the impact of targeted, context-specific interventions (29, 30).

Conversely, high-SDI countries and regions including Japan, Republic of Korea, Taiwan (Province of China), Singapore, and Belgium show significant gaps between their actual performance and what would be expected given their development status. These discrepancies may stem from incomplete vaccine uptake, gaps in adult screening programs, or lack of harm reduction services for key populations. This analysis challenges the assumption that economic development alone ensures disease control, and instead highlights the importance of policy prioritization and programmatic efficiency. It also opens a window for cross-context learning: for instance, lessons from Nepal’s hepatitis A vaccination rollout or Somalia’s hepatitis B immunization program could inform strategic refinements in high-income nations.

Perhaps most critically, the synthesis of these patterns reveals a broader insight: while the epidemiology of hepatitis is subtype-specific, the solutions must be integrated within a country’s public health system. Rather than tackling each subtype in isolation, health authorities should adopt a platform approach—leveraging vaccination infrastructure, water safety interventions, and harm reduction programs in a coordinated, synergistic manner. Moreover, the decomposition and frontier methods applied in this study not only elucidate current gaps but also offer a scalable monitoring framework for global hepatitis elimination targets. Future strategies must focus not only on reducing absolute incidence but also on maximizing efficiency and equity in health system responses.

### New insights and contributions to the literature

This study advances the current literature by providing an integrated, cross-subtype analysis of the global burden of acute viral hepatitis. While earlier investigations have typically focused on a single hepatitis subtype or limited their scope to specific regions or populations (31, 32), our study applies a harmonized analytical framework across hepatitis A, B, C, and E, enabling direct comparison of spatial distribution, temporal trends, and underlying determinants. This approach facilitates a more holistic understanding of how hepatitis epidemics manifest and evolve in varying sociogeographic contexts.

By combining spatial mapping, decomposition analysis, and frontier benchmarking, our research uncovers both common and subtype-specific patterns. The spatial analysis identifies clusters of high incidences that transcend individual hepatitis types, suggesting that certain structural vulnerabilities, such as inadequate infrastructure and weak health systems, create an enabling environment for multiple types of transmission. Meanwhile, the decomposition analysis provides a quantifiable assessment of the relative impact of population growth, demographic change, and epidemiological transitions, clarifying the mechanisms through which hepatitis burdens shift over time. The application of frontier benchmarking adds a new dimension to global hepatitis research by highlighting inefficiencies in disease control relative to national development status. This technique allows us to differentiate between countries where incidence remains high due to systemic limitations and those where the potential for improvement is significant but underutilized.

Collectively, these findings represent a substantial contribution to the field. The study moves beyond descriptive epidemiology by offering comparative insight across hepatitis subtypes and by proposing an evaluative framework that links health outcomes to achievable benchmarks. Such an approach provides a stronger empirical basis for assessing progress toward global hepatitis elimination goals and offers a replicable model for examining other infectious diseases with complex social and geographic dynamics.

### Policy implications

The results of this study underscore the need for policy approaches that are both context-sensitive and system-oriented. Addressing the global burden of acute hepatitis requires more than isolated technical solutions. It demands a coordinated strategy that aligns intervention design with the specific transmission patterns and structural determinants relevant to each hepatitis subtype.

In regions with a high incidence of hepatitis A and E, particularly in parts of Africa and India, the most urgent need is to strengthen water, sanitation, and hygiene systems. These improvements not only reduce the risk of fecal-oral transmission but also provide broader health co-benefits by preventing other enteric diseases. WASH investments should be coupled with efforts to expand hepatitis A vaccination, especially in areas with recurrent outbreaks and poor infrastructure.

In the case of hepatitis B and C, policy responses must focus on scaling up prevention and treatment among vulnerable and underserved populations. This includes improving birth-dose coverage for hepatitis B, expanding adult immunization where applicable, and ensuring the availability of antiviral therapies. Harm reduction programs such as sterile needle exchange and opioid substitution therapy remain critical for controlling hepatitis C in regions where injecting drug use is a key transmission route. However, these services continue to face social, legal, and financial barriers in many countries, which must be addressed through inclusive public health legislation and sustained funding.

High-income countries are not exempt from the challenges highlighted in this analysis. Despite advanced healthcare infrastructure, several of these nations perform below expectations relative to their development status. This finding points to inefficiencies in the implementation of screening programs, incomplete vaccine coverage among adults, and missed opportunities for integrating hepatitis services into primary care. Future policy frameworks in these settings should prioritize universal screening strategies, with a focus on marginalized groups such as migrants, incarcerated populations, and people who use drugs.

Ultimately, the study’s results emphasize the importance of adopting an integrated approach to hepatitis prevention and control. Interventions should be designed to function within existing health systems while also addressing the unique drivers of transmission in different settings. Policymakers must move beyond subtype-specific silos and recognize the value of coordinated efforts that simultaneously target multiple forms of viral hepatitis. Only through such an inclusive and responsive policy framework can global hepatitis reduction goals be meaningfully achieved.

## Conclusion

The uneven global burden of acute viral hepatitis revealed in this comprehensive analysis underscores the need for tailored, context-specific interventions. The stark geographical disparities and the distinct epidemiological drivers for each hepatitis subtype call for a nuanced approach that addresses the unique challenges faced by different regions and socioeconomic settings. By leveraging the frontier analysis, this study has identified countries with the greatest potential for further reductions in acute hepatitis incidence, highlighting both success stories and opportunities for improvement across the development spectrum. Moving forward, governments and public health agencies must prioritize investments in water, sanitation, and hygiene infrastructure, strengthen routine vaccination programs, and implement targeted harm reduction strategies to effectively mitigate the persistent burden of acute viral hepatitis worldwide. Integrating these context-specific interventions with a deeper understanding of the underlying drivers will be crucial in steering the global community towards a future with improved acute hepatitis outcomes.

## Data Availability

The original contributions presented in the study are included in the article/Supplementary material, further inquiries can be directed to the corresponding author.

## References

[1] Misumi I Mitchell JE Lund MM Cullen JM Lemon SM Whitmire JK. T cells protect against hepatitis a virus infection and limit infection-induced liver injury. J Hepatol. (2021) 75:1323–34. doi: 10.1016/j.jhep.2021.07.019, PMID: 34331968 PMC8604763

[2] Zheng S Zhang H Chen R Yan J Han Q. Pregnancy complicated with hepatitis B virus infection and preterm birth: a retrospective cohort study. BMC Pregnancy Childbirth. (2021) 21:513. doi: 10.1186/s12884-021-03978-0, PMID: 34273944 PMC8286565

[3] Perz JF Grytdal S Beck S Fireteanu AM Poissant T Rizzo E. Case-control study of hepatitis B and hepatitis C in older adults: do healthcare exposures contribute to burden of new infections? Hepatology. (2013) 57:917–24. doi: 10.1002/hep.25688, PMID: 22383058

[4] Anyiwe K Erman A Hassan M Feld JJ Pullenayegum E Wong WWL. Characterising the effectiveness of social determinants of health-focused hepatitis B interventions: a systematic review. Lancet Infect Dis. (2024) 24:e366–85. doi: 10.1016/S1473-3099(23)00590-X, PMID: 38184004

[5] Behrendt P Friesland M Wißmann JE Kinast V Stahl Y Praditya D. Hepatitis E virus is highly resistant to alcohol-based disinfectants. J Hepatol. (2022) 76:1062–9. doi: 10.1016/j.jhep.2022.01.006, PMID: 35085595

[6] Choi WM Yip TC Kim WR Yee LJ Brooks-Rooney C Curteis T. Chronic hepatitis B baseline viral load and on-treatment liver cancer risk: a multinational cohort study of HBeAg-positive patients. Hepatology. (2024) 80:428–39. 38436992 10.1097/HEP.0000000000000752 PMC11251501

[7] Kim GA Choi SW Han S Lim YS. Non-linear association between liver fibrosis scores and viral load in patients with chronic hepatitis B. Clin Mol Hepatol. (2024) 30:793–806. doi: 10.3350/cmh.2024.0252, PMID: 39026397 PMC11540400

[8] Brauer M Roth GA Aravkin AY Zheng P Abate KH Abate YH. Global burden and strength of evidence for 88 risk factors in 204 countries and 811 subnational locations, 1990-2021: a systematic analysis for the global burden of disease study 2021. Lancet. (2024) 403:2162–203. doi: 10.1016/S0140-6736(24)00933-4, PMID: 38762324 PMC11120204

[9] Cheng X Yang Y Schwebel DC Liu Z Li L Cheng P. Population ageing and mortality during 1990-2017: a global decomposition analysis. PLoS Med. (2020) 17:e1003138. doi: 10.1371/journal.pmed.1003138, PMID: 32511229 PMC7279585

[10] Das GP. Standardization and decomposition of rates from cross-classified data. Genus. (1994) 50:171–96. 12319256

[11] Lemoine M Eholié S Lacombe K. Reducing the neglected burden of viral hepatitis in Africa: strategies for a global approach. J Hepatol. (2015) 62:469–76. doi: 10.1016/j.jhep.2014.10.008, PMID: 25457207

[12] Zeng DY Li JM Lin S Dong X You J Xing QQ. Global burden of acute viral hepatitis and its association with socioeconomic development status, 1990-2019. J Hepatol. (2021) 75:547–56. doi: 10.1016/j.jhep.2021.04.035 33961940

[13] Fantilli AC Masachessi G Cola GD Castro G Sicilia P Marinzalda MLA. Integrated hepatitis e virus monitoring in Central Argentina: a six-year analysis of clinical surveillance and wastewater-based epidemiology. Water Res. (2024) 261:122004. 38991242 10.1016/j.watres.2024.122004

[14] Marti M Macartney K Grais RF Aggarwal R. Hepatitis E vaccination: continued benefit for pregnant women in vulnerable settings. Lancet Glob Health. (2024) 12:e1758. doi: 10.1016/S2214-109X(24)00290-0 39032497

[15] Van Damme P Pintó RM Feng Z Cui F Gentile A Shouval D. Hepatitis a virus infection. Nat Rev Dis Primers. (2023) 9:51. doi: 10.1038/s41572-023-00461-2, PMID: 37770459

[16] Hartard C Gantzer C Bronowicki JP Schvoerer E. Emerging hepatitis E virus compared with hepatitis a virus: a new sanitary challenge. Rev Med Virol. (2019) 29:2078. 10.1002/rmv.2078 31456241

[17] Huang S Zhang X Su Y Zhuang C Tang Z Huang X. Long-term efficacy of a recombinant hepatitis E vaccine in adults: 10-year results from a randomised, double-blind, placebo-controlled, phase 3 trial. Lancet. (2024) 403:813–23. doi: 10.1016/S0140-6736(23)02234-1, PMID: 38387470

[18] Kirkwood CD Dobscha KR Steele AD. Hepatitis E should be a global public health priority: recommendations for improving surveillance and prevention. Expert Rev Vaccines. (2020) 19:1129–40. doi: 10.1080/14760584.2020.1874930, PMID: 33441054

[19] Rosenthal P. Cost-effectiveness of hepatitis a vaccination in children, adolescents, and adults. Hepatology. (2003) 37:44–51. doi: 10.1053/jhep.2003.50016, PMID: 12500187

[20] Das A. An economic analysis of different strategies of immunization against hepatitis a virus in developed countries. Hepatology. (1999) 29:548–52. doi: 10.1002/hep.510290225, PMID: 9918934

[21] Sadio AJ Ferré VM Adama OIW Kouanfack HR Dagnra AC Amenyah-Ehlan AP. Street adolescents in low income setting exposed to hepatitis B and C, and disadvantaged by lifestyle: a Togolese cross-sectional study. BMC Public Health. (2024) 24:1901. doi: 10.1186/s12889-024-19415-8, PMID: 39014371 PMC11250937

[22] Artenie A Stone J Fraser H Stewart D Arum C Lim AG. Incidence of HIV and hepatitis C virus among people who inject drugs, and associations with age and sex or gender: a global systematic review and meta-analysis. Lancet Gastroenterol Hepatol. (2023) 8:533–52. doi: 10.1016/S2468-1253(23)00018-3, PMID: 36996853 PMC10817215

[23] Yin S Ly KN Barker LK Bixler D Thompson ND Gupta N. Estimating the prevalence of injection drug use among acute hepatitis C cases from a National Surveillance System: application of random Forest-based multiple imputation. J Public Health Manag Practice. (2024) 30:733–43. doi: 10.1097/PHH.0000000000002014, PMID: 39041767 PMC11883639

[24] Tian F Forouzannia F Feng Z Biondi MJ Mendlowitz AB Feld JJ. Feasibility of hepatitis C elimination by screening and treatment alone in high-income countries. Hepatology. (2024) 80:440–50. doi: 10.1097/HEP.0000000000000779, PMID: 38478751 PMC11251502

[25] Patterson J Abdullahi L Hussey GD Muloiwa R Kagina BM. A systematic review of the epidemiology of hepatitis a in Africa. BMC Infect Dis. (2019) 19:651. doi: 10.1186/s12879-019-4235-5, PMID: 31331281 PMC6647100

[26] Kanyenda TJ Abdullahi LH Hussey GD Kagina BM. Epidemiology of hepatitis a virus in Africa among persons aged 1-10 years: a systematic review protocol. Syst Rev. (2015) 4:129. doi: 10.1186/s13643-015-0112-5, PMID: 26419360 PMC4589083

[27] Ali AS Hussein NA Elmi EOH Ismail AM Abdi MM. Hepatitis B vaccination coverage and associated factors among medical students: a cross-sectional study in Bosaso, Somalia, 2021. BMC Public Health. (2023) 23:1060. doi: 10.1186/s12889-023-15992-2, PMID: 37268892 PMC10239102

[28] Hussein NA Ismail AM Jama SS. Assessment of hepatitis B vaccination status and associated factors among healthcare Workers in Bosaso, Puntland, Somalia 2020. Bio Med Res Int. (2022) 2022:9074294. doi: 10.1155/2022/9074294, PMID: 35355823 PMC8960009

[29] Khadka S Pandit R Dhital S Baniya JB Tiwari S Shrestha B. Evaluation of five international HBV treatment guidelines: recommendation for resource-limited developing countries based on the national study in Nepal. Pathophysiology. (2020) 27:3–13. doi: 10.3390/pathophysiology27010002 34321716 PMC8315108

[30] Vincent JP Nyamasege C Wang S Madec Y Shimakawa Y. Prevalence of hepatitis B, C, and D virus infection in Haiti: a systematic review and meta-analysis. Front Public Health. (2022) 10:1099571. doi: 10.3389/fpubh.2022.1099571, PMID: 36711383 PMC9874305

[31] Huang D Lai H Shi X Jiang J Zhu Z Peng J. Global temporal trends and projections of acute hepatitis E incidence among women of childbearing age: age-period-cohort analysis 2021. J Infect. (2024) 89:106250. doi: 10.1016/j.jinf.2024.106250, PMID: 39181413

[32] Banach M. Global, regional, and national burden of hepatitis B, 1990-2019: a systematic analysis for the global burden of disease study 2019. Lancet Gastroenterol Hepatol. (2022) 7:796–829. doi: 10.1016/S2468-1253(22)00124-8, PMID: 35738290 PMC9349325

