# Supplementary material for: Geospatial patterns and socioeconomic determinants of the global acute viral hepatitis burden
Source: Front Public Health. 2025 Jun 5;13:1581484. doi: 10.3389/fpubh.2025.1581484 (PMC12176882; doi:10.3389/fpubh.2025.1581484)
Supplement: Supplementary file 2 [file Supplementary_file_2.DOCX]

**Supplementary Methods**

**Decomposition analysis:**

We first used the decomposition methodology of Das Gupta^1-3^ to decompose Incidence rate by population age structure, population growth, and epidemiologic changes. The number of Incidence rate at each location was obtained from the following formula:

INCIDENCE RATE _ay, py, ey_ = $\sum_{i=1}^{20} ($a _i, y_ * p _y_ * e _i, y_)

Where INCIDENCE RATE _ay, py, ey_ represented Incidence rate based on the factors of age structure, population, and Incidence rate rate for specific year y; a _i_ _y_ represents the proportion of population for the age category i in given year y; p _y_ represents the total population in given year y; and e _i, y_ represents Incidence rate rate given age category i in year y. The contribution of each factor to the change in Incidence rate from 1990 to 2021 was defined by the effect of one factor changing while the other factors were held constant. For example, the effect of age structure was calculated as:

[(INCIDENCE RATE _a2021, p1990, e1990_ + INCIDENCE RATE _a2021, p2021, e2021_)/3+ (INCIDENCE RATE _a2021, p1990, e2021_ + INCIDENCE RATE _a2021, p2021, e1990_)/6] - [(INCIDENCE RATE _a1990, p2021, e2021_ + INCIDENCE RATE _a1990, p1990, e1990_)/3+ (INCIDENCE RATE _a1990, p2021b, e1990_ + INCIDENCE RATE _a1990, p1990, e2021_)/6]

**Frontier Analysis:**

To assess the relationship between burden and socio-demographic development, we utilized frontier analysis as a quantitative approach to identify the optimal achievable age-standardized Disability-Adjusted Life Years (Incidence rate) rates based on development status indicated by the Socio-demographic Index (SDI). The Incidence rate frontier represents the minimum Incidence rate that each country or territory could realistically achieve given its SDI. The distance from this frontier, referred to as the effective difference, indicates potential unrealized gains; a significant effective difference suggests opportunities for reducing Incidence rate that could be attainable based on the country's development level. We employed a data envelope analysis, which facilitates the definition of non-linear frontiers, using the free disposal hull method to create a frontier for age-adjusted Incidence rate across different SDI values, utilizing data from 1990 to 2021. To account for uncertainty, we generated 1,000 bootstrapped samples, randomly selecting with replacement from all countries and territories over the years. We then calculated the mean Incidence rate for each SDI value from these bootstrapped samples. A LOESS regression with a local polynomial degree of 1 and a span of 0.2 was applied to smooth the frontier. To mitigate the influence of outliers, countries with super-efficient Incidence rate were excluded from the frontier generation. In analyzing the relationship of age-standardized Incidence rate rates in relation to the frontier for the year 2021, we computed the effective difference by measuring the absolute distance from the frontier using the 2021 SDI and age-standardized Incidence rate data for each country or territory. Any countries or territories with Incidence rate lower than the frontier were assigned a distance of zero^4,5^.

**References:**

1 P., D. G. *Standardization and decomposition of rates: a user’s manual, Pages 19-36*. 19-36 (1993).

2 Das Gupta, P. Standardization and decomposition of rates from cross-classified data. *Genus* **50**, 171-196 (1994).

3 Chevan, A. & Sutherland, M. Revisiting Das Gupta: refinement and extension of standardization and decomposition. *Demography* **46**, 429-449 (2009).

4 Access, G. B. D. H., Quality Collaborators. Electronic address, c. u. e., Access, G. B. D. H. & Quality, C. Healthcare Access and Quality Index based on mortality from causes amenable to personal health care in 195 countries and territories, 1990-2015: a novel analysis from the Global Burden of Disease Study 2015. *Lancet*, doi:10.1016/S0140-6736(17)30818-8 (2017).

5 Xie, Y., Bowe, B., Xian, H., Balasubramanian, S. & Al-Aly, Z. Rate of Kidney Function Decline and Risk of Hospitalizations in Stage 3A . *Clinical journal of the American Society of Nephrology : CJASN* **10**, 1946-1955, doi:10.2215/CJN.04480415 (2015).
